# Antigen vs RT-PCR Tests for Screening Quarantined Students in Florida During the COVID-19 Pandemic SARS-CoV-2 Delta Variant Surge

**DOI:** 10.1001/jamapediatrics.2022.0080

**Published:** 2022-03-07

**Authors:** Eric J. Nelson, Sarah Lindley McKune, Kathleen A. Ryan, Jerne Shapiro, Adrienne H. Mott-Young, Paul D. Myers, J. Glenn Morris

**Affiliations:** 1 Department of Pediatrics, University of Florida College of Medicine, Gainesville; 2 Department of Environmental and Global Health, University of Florida College of Public Health and Health Professions, Gainesville; 3 Department of Epidemiology, University of Florida College of Public Health and Health Professions, Gainesville; 4 Florida Department of Health in Alachua, Gainesville; 5 Emerging Pathogens Institute, University of Florida, Gainesville

## Abstract

This diagnostic/prognostic study compares the results of antigen vs real-time reverse transcription–polymerase chain reaction tests among quarantined students 5 days after exposure to SARS-CoV-2 during the surge of Delta variant cases in the COVID-19 pandemic.

In the US, schools opened in fall 2021 during a surge of COVID-19 cases attributed to the SARS-CoV-2 Delta variant.^1^ The US Centers for Disease Control and Prevention recommended masking in schools and return to school of asymptomatic quarantined close contacts at day 7 after the last date of SARS-CoV-2 exposure with a negative test result or at day 10 without a test. Rapid antigen tests have been proposed as a tool to reduce or even eliminate quarantine,^2^ but there are uncertainties about their performance compared with real-time reverse transcription–polymerase chain reaction (RT-PCR) tests.^3,4,5^ Most validation studies on these tests were done before the Delta variant emerged, which may impact results because of the variant’s higher viral load.^6^ The aim of this study was to assess whether rapid antigen and RT-PCR tests gave comparable results and whether antigen testing on day 5 after SARS-CoV-2 exposure would be helpful in making decisions about when quarantined children can return to school.

## Methods

In this diagnostic/prognostic study, data were collected from August 23, to October 6, 2021, in Florida in Alachua County’s 38 public schools that serve a population of 29 541 K-12 students. Data on race and ethnicity were not collected because they were not essential to the primary study objective. During the study period, masks were mandated for all students unless they had a physician-signed medical exemption. In an effort to reduce school days lost, the Florida Department of Health (FDOH) mandated that asymptomatic quarantined students be allowed to return to school at day 5 after exposure with a test result negative for SARS-CoV-2 or at day 8 without a test. This study was evaluated by the FDOH and was deemed exempt from review under 45 CFR 46.102(l)(2) because it was a public health surveillance activity. Written informed consent was obtained from students’ parents before sample collection.

The FDOH in Alachua County provided free rapid antigen (BinaxNow; Abbott) and concurrent RT-PCR testing for students 5 days after the last date of exposure to SARS-CoV-2 at school. Two anterior nasal swabs were obtained from each student—one for RT-PCR testing and the other for rapid antigen testing on site. The percentages of quarantined students with positive rapid antigen and RT-PCR test results were enumerated. Congruence analysis was performed using R software, version 3.5.1 (R Foundation for Statistical Computing) and RStudio, version 1.1.463 (RStudio, PBC).

## Results

Between August 23 and October 6, 2021, the average SARS-CoV-2 test positivity rate in Alachua County was 11.7% across all ages and 10.8% for school-aged children (aged 5 to 19 years). Among isolates from Alachua County sequenced at the University of Florida, 95.9% were the Delta variant. Of 29 541 students, 1731 symptomatic students were known to have been tested, and 1601 had a positive test result for SARS-CoV-2, representing 5.4% of all students in the county (Figure). Of 1036 asymptomatic quarantined school contacts of the students with positive test results, 603 (58.2%) had paired samples collected on day 5 after exposure. Of these 603 student contacts tested, 25 (4.1%) had a positive rapid antigen test result (independent of RT-PCR test results), and 30 (5.0%) had a positive RT-PCR test result (independent of rapid antigen test results). Taken in concert, 24 (4.0%) had positive test results by both modalities, 1 (0.2%) had positive antigen and negative RT-PCR results, 6 (1.0%) had negative antigen and positive RT-PCR results, and 572 (94.9%) had negative results by both modalities (rapid antigen sensitivity, 80.0% [95% CI, 61.4%-92.3%]; specificity, 99.8% [95% CI, 99.0%-100.0%]). The tests were highly concordant (Cohen κ, 0.87 [95% CI, 0.79-0.95]) (Table).

**Figure.**
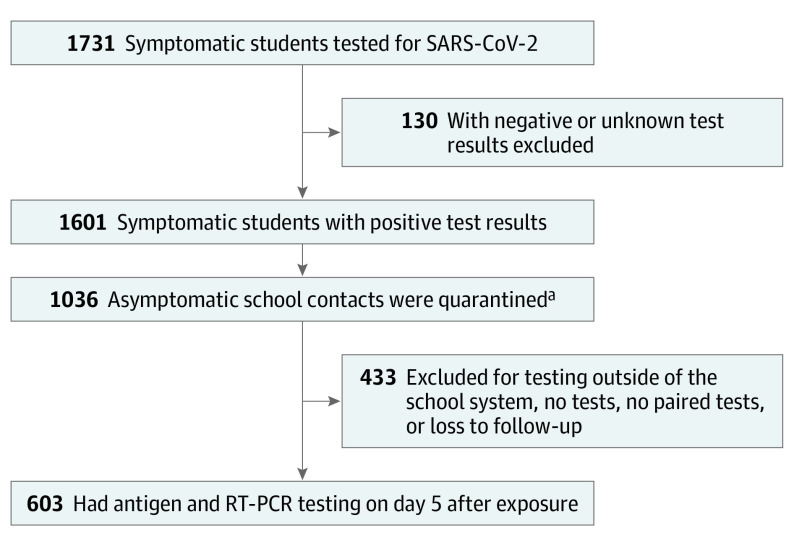
Sample Collection Diagram ^a^Students with test results positive for SARS-CoV-2 often had their testing performed outside of the school system. Although these students are included in the case number total, some of them did not generate school-based quarantined contacts because of an exemption owing to vaccination or previous SARS-CoV-2 infection or because testing outside the school system failed to trigger timely contact tracing. RT-PCR indicates reverse transcription-polymerase chain reaction.

**Table.** Concordance Analysis of Rapid Antigen and RT-PCR Tests Among K-12 Students

Age group, y	Positive Ag and RT-PCR test results (%)	Positive Ag and negative RT-PCR test results (%)	Negative Ag and positive RT-PCR test results (%)	Negative Ag and RT-PCR test results (%)	Total
All	24 (4.0)	1 (0.2)	6 (1.0)	572 (94.9)	603^a^
5-10	10 (3.2)	1 (0.3)	2 (0.6)	301 (95.9)	314^b^
11-15	13 (6.2)	0	2 (0.9)	196 (92.9)	211^c^
16-19	1 (1.3)	0	2 (2.6)	75 (96.2)	78^d^

Abbreviations: Ag, antigen; RT-PCR, reverse transcription–polymerase chain reaction.

^a^ Cohen κ, 0.87 (95% CI, 0.79-0.95); sensitivity, 80.0% (95% CI, 61.4%-92.3%); specificity, 99.8% (95% CI, 99.0%-100.0%); positive predictive value (PPV), 96.0% (95% CI, 79.7%%-99.9%); negative predictive value (NPV), 99.0% (95% CI, 97.8%-99.6%).

^b^ Cohen κ, 0.86 (95% CI, 0.75-0.98); sensitivity, 83.3% (95% CI, 51.6%-97.9%); specificity, 100.0% (95% CI, 98.0%-100.0%); PPV, 90.9% (95% CI, 58.7%-99.8%); NPV, 99.3% (95% CI, 97.6%-99.9%).

^c^ Cohen κ, 0.92 (95% CI, 0.79-1.05); sensitivity, 86.7% (95% CI, 59.5%-98.3%); specificity, 100.0% (95% CI, 98.1%-100.0%); PPV, 100% (95% CI, 75.3%-100.0%); NPV, 99.0% (95% CI, 96.4%-99.9%).

^d^ Cohen κ, 0.49 (95% CI, 0.30-0.68); sensitivity, 33.3% (95% CI, 0.8%-90.6%); specificity, 100.0% (95% CI, 95.2%-100.0%); PPV, 100% (95% CI, 2.5%-100.0%); NPV, 97.4% (95% CI, 90.9%-99.7%).

## Discussion

In this diagnostic/prognostic study, we evaluated the use of rapid antigen testing at day 5 after exposure among asymptomatic quarantined school contacts of students with confirmed SARS-CoV-2 test results in 1 Florida county during a surge of cases attributed to the Delta variant in the COVID-19 pandemic. The rapid antigen test was highly congruent with the reference of RT-PCR from a nasal swab. This study was conducted in parallel with a policy of immediate return to school if the rapid antigen test result was negative. Through self-reported passive surveillance, no SARS-CoV-2 infections were identified among students who returned to school during the study period. The high congruence of the rapid antigen test with RT-PCR test may inform the recommendations to minimize student absenteeism owing to quarantine.

This study has limitations. Detection of infection among students was based on passive surveillance alone using volunteered symptoms or test positivity. Also, the rates of school contacts quarantined and the percentage of contacts sampled were low compared with the respective totals.
